# Tuberculosis of the breast

**DOI:** 10.4103/1817-1737.41918

**Published:** 2008

**Authors:** Salim Baharoon

**Affiliations:** *Department of Medicine, Division of Infectious Diseases, King Abdulaziz Medical City, King Fahad National Guard Hospital, Riyadh, Kingdom of Saudi Arabia*

**Keywords:** Tuberculous mastitis (TM), Direct amplification tests (DAT), M tuberculosis direct test (MTD)

## Abstract

Tuberculosis of the breast is an uncommon disease even in countries where the incidence of pulmonary and extrapulmonary tuberculosis is high. Clinical presentation is usually of a solitary, ill-defined, unilateral hard lump situated in the upper outer quadrant of the breast. This disease can present a diagnostic problem on radiological and microbiological investigations, and thus a high index of suspicion is needed. Incorporating a highly sensitive technique like polymerase chain reaction (PCR) may be helpful in establishing the usefulness of such technology and can aid in conforming the diagnosis early. The disease is curable with antitubercular drugs, and surgery is rarely required

## Introduction

Tuberculosis is the most widespread and persistent human infection in the world. The infection can involve any organ and mimic other illness, hence it is called the great mimicker.

Tuberculosis of the breast is an uncommon presentation of tuberculosis, even in countries where the incidence of pulmonary and extrapulmonary tuberculosis is high.[1,2] We will review the current knowledge of this rare manifestation of a common disease.

## Incidence

Tuberculous mastitis (TM) is a rare extrapulmonary presentation of tuberculosis accounting for less than 1% of all diseases of the breast in the industrialized world.[1,2–5] Incidence of this disease is higher in countries endemic for tuberculosis, like the Indian subcontinent, where it may be as high as 4%.[2] In the Arabian Gulf, the frequency of the disease is reported to be between 0.4% and 0.5%.[6,7]

Sir Astley Cooper reported the first case of tuberculous mastitis in 1829 and called it ‘scrofulous swelling of the bosom.’[8] TM may be part of a systemic disease or may be the only manifestation of tuberculosis. It occurs far more frequently in women, especially in their reproductive age, and is uncommon in prepubescent and elderly women.[9,10] This parallels the highest incidence of pulmonary tuberculosis.[11] This could be because the female breast undergoes frequent changes during the period of childbearing activity and is more susceptible to trauma and infection.[12]

The disease is very rare in males; in a review by Gupta *et al.* comprising 160 patients, only 6 were males.[13] The risk factors associated with TM include multiparity, lactation, trauma, past history of suppurative mastitis, and AIDS.[13,14] Patients will frequently be symptomatic for at least a few months prior to diagnosis.[12,15] It may be difficult to differentiate from carcinoma breast, a condition with which it may coexist.[16,17]

## Clinical Presentation

The most common clinical presentation of tuberculous mastitis is that of a solitary, ill-defined, unilateral hard lump situated in the central or upper outer quadrant.[9,18,19] The lesion may be indistinguishable from carcinoma breast, being irregular, hard, and at times, fixed to either skin or muscle or even chest wall.[19] Multiple lumps and bilateral involvement are uncommon and occur in less than 3% of the patients.[18,20] The lesion may progress to a tuberculous ulcer over the breast skin and tuberculous breast abscess with or without discharging sinuses.[11] In a series of 30 patients recently reported by Tewari, 22 patients presented with lump in the breast; 11 of these had tubercular ulcer, and 4 had multiple discharging sinuses in the overlying breast skin.[21]

One third of the patients have breast pain with or without increased breast nodularity, and one third have ipsilateral axillary lymph node involvement.[9,19] Pulmonary involvement occurs only rarely.[9] Another form of presentation in recent years is tuberculous breast abscess.[21,22] This form is described to be more prevalent in endemic areas of TB and presents usually in young females.

## Classification of Breast Tuberculosis

Mckeown *et al.*[23] classified TM into 5 pathological varieties. The nodular form is the most common variety and usually presents as a localized slowly growing mass that progresses to involve skin, may ulcerate, and can form sinuses. Histologically, this form is characterized by extensive caseation and little fibrosis.[12,15,24]

The diffuse or disseminated form is the second most common variety and involves the entire breast with multiple intercommunicating foci of tubercles within the breast, which caseate leading to ulceration and discharging sinuses. The overlying skin is thickened with multiple ulcers. Ipsilateral axillary lymph nodes are usually enlarged and matted. This form is more common in older females and may be confused with malignancy.[22]

The third type described by Mckeown is the sclerosing form. This variety demonstrates extensive fibrosis rather than caseation, in which the entire breast is hard and the nipple is retracted. This form is often seen in involuting breasts of older females and may be also mistaken for carcinoma breast.[23]

The last two forms described by Mckeown are tuberculous mastitis obliterans and acute miliary tuberculous mastitis. Tuberculous mastitis obliterans is characterized by duct infection producing proliferation of lining epithelium and marked epithelial and periductal fibrosis. The ducts are occluded and cystic spaces are produced resembling ‘cystic mastitis.’ Acute miliary tuberculous mastitis occurs as a part of generalized miliary tuberculosis. Both forms are rarely encountered in recent literature and may be of historical importance only.

Tewari has recently suggested reclassifying breast tuberculosis into 3 categories, namely, nodular, disseminated, and abscess varieties.[21] The new classification takes into consideration the changes seen in clinical presentation of tuberculosis over the last two decades. Sclerosing tubercular mastitis, tuberculous mastitis obliterans, and acute miliary tubercular mastitis are all very rare today, while tuberculous breast abscess is more frequent. The latter is common among young females and represents up to 30% of cases in recent publications.[21,22]

## Routes of Infection

Tuberculous involvement of breast occurs either by direct inoculation of the bacilli through abrasions in the nipple, which is rare[13,19]; or more commonly via lymphatic, hematogenous, or contiguous seeding.[25,26] The lymphatic route is the most likely route of breast involvement which occurs by retrograde extension from the axillary lymph node. This hypothesis is supported by the involvement of axillary nodes, frequently ipsilateral nodes, in 50% to 75% of tuberculous mastitis cases.[15,19]

Contiguous spread occurs from the ribs, pleural space, or rectus sheath from an intra-abdominal source.[13,27] Hematogenous spread is rare and occurs in cases of disseminated tuberculosis.

## Diagnostic Strategies

The gold standard diagnosis of TM is by bacteriological culture of breast tissue or by Ziehl Neelsen (ZN) stain.[13] However, in TM the bacilli are isolated in only 25% of cases, and acid-fast bacilli (AFB) are identified only in 12% of the patients. Therefore, demonstration of caseating granulomas from the breast tissue and involved lymph nodes may be sufficient for the diagnosis.[21,27,28] Fine needle aspiration (FNAC) is the most widely used initial invasive method for diagnosis of breast tuberculosis. Approximately 73% of the cases of TM can be diagnosed on FNAC when both epithelioid cell granulomas and necrosis are present.[5,29] In tuberculosis-endemic countries, the finding of granuloma in fine needle aspiration warrants empirical treatment for tuberculosis even in the absence of positive acid fast bacilli (AFB) and without culture results.[5,30]

An excision biopsy is strongly advocated, however, to rule out other diagnoses like sarcoidosis, fungal infections, ductular ectasia, and a coexisting malignancy.[16,21,31,28,32] Adequate tissue samples are not usually possible with fine needle aspiration.[29] Distinguishing idiopathic granulomatous mastitis from tuberculous mastitis is extremely important as treatment options of the former may include steroids. Steroids may flare up tuberculosis; and in tuberculosis-endemic regions, empiric antituberculous therapy may be warranted before considering steroids therapy. Table 1 illustrates some distinguishing clinical and histological features of tuberculous mastitis and idiopathic granulomatous mastitis.

Since the detection of AFB in a smear requires more than 10,000 organisms/mL, nucleic acid amplification test could be very helpful in establishing the diagnosis of TB in smear-negative samples. With the use of amplification systems, nucleic acid sequences unique to *Mycobacterium tuberculosis* (*M. tuberculosis*) can be detected directly from clinical samples, offering better accuracy than acid fast bacilli (AFB) smear and greater speed than culture. Two direct amplification tests (DATs) have been approved by the FDA, the *M. tuberculosis* direct test (MTD; Gen-Probe, San Diego, CA) and the Amplicor *M. tuberculosis* test (AMPLICOR MTB Test; Roche Diagnostic Systems, Branchburg, NJ). Both tests amplify and detect *M. tuberculosis* 16S ribosomal RNA.[33,34]

The appropriate use of these DATs in diagnosis of tuberculosis has yet to be completely determined.[35,36] The specificity of DAT approaches 100% and sensitivity is about 96% in AFB smear-positive specimens, and diagnosis of pulmonary TB can be established if the two are present.[37,38] However, in AFB smear-negative samples, the specificity, sensitivity, and positive predictive value vary significantly with the pretest probability of the disease.[38] Catanzaro *et al.* found that the positive predictive value of *M. tuberculosis* direct test in a pulmonary sample approaches 100% if pulmonary tuberculosis is strongly clinically suspected but is only 59% with low clinical suspicion. The negative predictive value, however, of a negative PCR is about 91% even if pulmonary TB is strongly suspected, in contrast to a value of 37% for AFB stain in the same clinical setting.[34] Similar results were found by Cohen RA *et al.*, who reported a sensitivity of 53% and a specificity of 93% of DAT versus culture when the Amplicor assay was applied to smear-negative specimens.[39] So a positive DAT result may still be valuable in the early detection of the approximately 50% of active tuberculosis cases which are smear negative.[39]

Several reports have described the use of DATs on nonrespiratory specimens, including lymph nodes, CSF, gastric fluid, bronco-alveolar lavage, and skin biopsies[34,40–44] and have reported high sensitivity, specificity, and positive and negative predictive values (sensitivity, 86%; specificity, 100%; positive predictive value, 100%; negative predictive value, 90%).[45,46] Shah *et al.* performed AFB smear, AFB culture, and the DAT (Amplicor assay) on 1090 tissue and body fluid specimens. They found PCR test to be very useful for detecting *M. tuberculosis* in nonrespiratory samples, which have lower frequency of positive AFB smear. When PCR test results were compared with the confirmed clinical diagnosis of tuberculosis, the sensitivity, specificity, positive predictive value, and negative predictive value for the PCR test were 76.4%, 99.8%, 92.8%, and 99.2% respectively.[45,47]

The role of polymerase chain reaction (PCR) in the diagnosis of breast tuberculosis, however, is less often reported.[48] Recently Khurram *et al.* reported a case series of 22 patients diagnosed with breast carcinoma with an associated granulomatous reaction in axillary lymph nodes with or without necrosis.[49] All samples were examined using ZN stain for AFB and nested PCR assays for *M. tuberculosis* DNA. In all the cases, ZN stains for AFB were negative. *M. tuberculosis* DNA was detected in 11 (50%) out of the 22 cases. Six of 12 cases which had granulomas in association with necrosis were positive for MTB-DNA, while 5 of 10 cases without necrosis were also positive for MTB-DNA. The authors did not however report that on how many patients were the cultures positive for MTB.

Given the high rate of AFB-negative stains in breast tissue and the overlap of clinical presentation [Table 1], direct amplification tests may serve as a valuable tool for diagnosis of breast tuberculosis. As treatment of other conditions that may be confused with tuberculous mastitis can potentially lead to dissemination of disease (steroids and methotrexate for idiopathic granulomatous mastitis), relying on procedures like FNAC and histopathology alone is not adequate in our view. This is particularly true in countries endemic with TB or for patients belonging to any high-risk group, like immigrants from endemic areas.[50] Tuberculous mastitis should seriously be considered in such clinical settings, and MTB-PCR should be part of the investigation requested in clinical samples from breast tissues.[50]

**Table 1:** Distinguishing features of tuberculous mastitis and idiopathic granulomatous mastitis

Granulomatous Mastitis		

	Idiopathic granulomatous mastitis	Tuberculous mastitis
Clinical	Appears after pregnancy	No relation to pregnancy
	No constitutional symptoms	Constitutional symptoms present
	No relation to breast feeding	No relation to breast feeding
	Possible relation with oral pills	No relation with oral pills
	Age 17 - 42 years	Any age
	Parous patients	Parous and Nonparous
	Hard mass, any site of breast but spare subareolar area	Hard mass any site of breast
	Bilateral is uncommon	Bilateral is common
	Rare nipple discharge	Occasional nipple discharge
	Tenderness present	Tenderness rare
	Rare axillary LN enlargement	Axillary LN can be enlarged
	Size of mass 1 - 8 cm	Size of mass 1 - 8 cm
	Clinically and Radiologically mimics carcinoma	Clinically and Radiologically mimics carcinoma
	**Idiopathic**	**Tuberculosis**
Histology	Lobules of Breast are affected	Any component of Breast tissue is affected (lobules, ducts and fat)
	Granulomas within the lobules	Granulomas anywhere
	Granuloma composed of Histiocytes, Langhans giant cells, lymphocytes, plasma cells and occasional eosinophilis	Granuloma composed of Histiocytes, Langhans giant cells, lymphocytes, rare plasma cells and eosinophilis
	Caseation necrosis absent	Cascation necrosis present
	Fat necrosis	Fat necrosis
	Fibrosis	Fibrosis
	Abscess common	Abscess uncommon

Radiological tools like mammography, computed tomography (CT scan), and magnetic resonance imaging (MRI) of the breast have all been used in diagnostic work-up of breast lumps. Either mammography or ultrasound of the breast may demonstrate a dense sinus tract connecting an ill-defined breast mass to a localized skin thickening. This ‘sinus tract sign,’ originally described by Makanjuola, may be strongly suggestive of tuberculous breast abscess but is found in only a small percentage of patients.[51,52]

**Figure 1:**
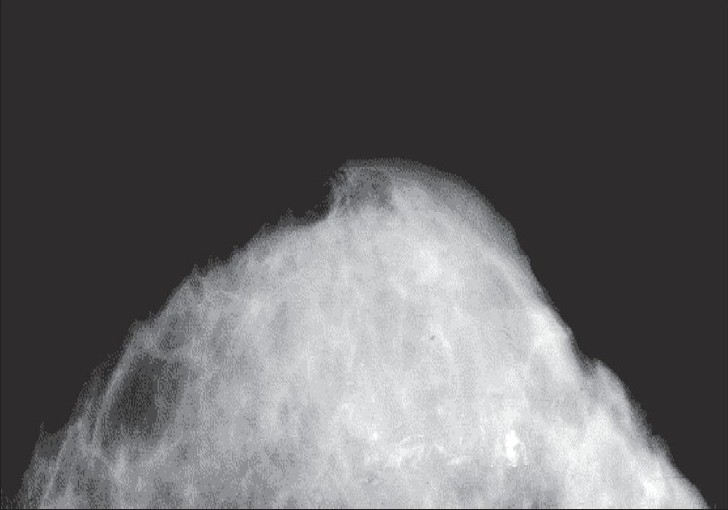
Distorted parenchyma with contracted Cooper's ligament and the so-called skin sinus and bulge sign

Radiological tools are generally helpful in defining the extent of the lesion but not very helpful in differentiating tuberculosis from other differential diagnoses, for example, malignancy [Figure 1].[53]

## Treatment

Medical therapy is the mainstay of therapy with antituberculous therapy (ATT). No specific guidelines are available for chemotherapy of breast tuberculosis, and therapy generally follows guidelines used for pulmonary tuberculosis. Success rate of medical therapy approaches 95% in most series with 6 months of antituberculous therapy (2 months of Isoniazid, Rifampicin, Pyrazinamide, and Ethambutol/4 months of Isoniazid and Rifampicin).[9,19] Some authors prefer the 9-month regimen (2 months of Isoniazid, Rifampicin, Pyrazinamide, and Ethambutol/7 months of Isoniazid and Rifampicin) due to lower relapse rate in general. Infection with multidrug-resistant tuberculosis (MDR) has been reported. Therapy with combination of first-line and second-line drugs that include kanamycin, ofloxacin, ethionamide, para-amino salicylic acid (PAS), pyrazinamide, and isoniazid has to be used.[54]

Surgical intervention was needed in up to 14% of the patients in some series, either due to lack of response to chemotherapy or large painful ulcerative lesions involving the entire breast.[21,55] Drainage of cold abscess in the axilla and breast to prevent sinus formation is mandatory. Axillary dissection may be required in patients with large ulcerated nodes.

Simple mastectomy is rarely needed nowadays and is reserved for patients with extensive disease comprising large painful ulcerated mass involving the entire breast and draining axillary lymph nodes.

## Conclusion

Tuberculosis of the breast is uncommon even in countries where the incidence of pulmonary and extrapulmonary tuberculosis is high.

This disease can present a diagnostic problem on radiological and microbiological investigations, and thus a high index of suspicion is needed. Incorporating a highly sensitive technique like PCR may be helpful in establishing the usefulness of such technology and can aid in conforming the diagnosis early. The disease is curable with antitubercular drugs, and surgery is rarely required.

## References

[1] Harris SH, Khan MA, Khan R, Haque F, Syed A, Ansari MM (2006). Mammary tuberculosis: Analysis of thirty-eight patients. ANZ J Surg.

[2] Tse GM, Poon CS, Ramachandram K, Ma TK, Pang LM, Law BK (2004). Granulomatous mastitis: A clinicopathological review of 26 cases. Pathology.

[3] Morgan M (1931). Tuberculosis of the breast. Surg Gynecol Obstet.

[4] Kalac N, Ozkan B, Bayiz H, Dursun AB, Demirag F (2002). Breast tuberculosis. Breast.

[5] Kakkar S, Kapila K, Singh MK, Verma K (2000). Tuberculosis of the breast: A cytomorphologic study. Ada Cytol.

[6] Al-Marri MR, Almosleh A, Almoslmani Y (2000). Primary tuberculosis of the breast in Qatar: Ten year experience and review of the literature. Eur J Surg.

[7] Oh KK, Kim JH, Kook SH (1998). Imaging of tuberculous disease involving breast. Eur Radiol.

[8] Cooper A, Longmans, Orme, Brown, Green (1829). Illustration of the diseases of the breast. Part I.

[9] Jalali U, Rasul S, Khan A, Baig N, Khan A, Akhter R (2005). Tuberculous mastitis. J Coll Physicians Surg Pak.

[10] O'Reilly M, Patel KR, Cummins R (2000). Tuberculosis of the breast presenting as carcinoma. Mil Med.

[11] Jaideep C, Kumar M, Klianna AK (1997). Male breast tuberculosis. Postgrad Med J.

[12] Raw N (1924). Tuberculosis of the breast. Br Med J.

[13] Gupta PP, Gupta KB, Yadav RK, Agarwal D (2003). Tuberculous mastitis: A review of seven consecutive cases. Indian J Tub.

[14] Gilbert AI, McGough EC, Farrell JJ (1962). Tuberculosis of the breast. Am J Surg.

[15] Mukerjee P, George M, Maheshwari HB, Rao CP (1974). Tuberculosis of the breast. J Indian Med Assoc.

[16] Fujii T, Kimura M, Yanagita Y, Koida T, Kuwano H (2003). Tuberculosis of axillary lymph nodes with primary breast cancer. Breast Cancer.

[17] Graunsman RI, Goldman ML, Graunsman RI, Goldman ML (1945). Tuberculosis of the breast-report of nine cases including two cases of co-existing carcinoma and tuberculosis. Am J Surg.

[18] Banerjee A, Green B, Burke M (1989). Tuberculous and granulomatous mastitis. Practitioner.

[19] Shinde SR, Chandawarkar RY, Deshmukh SP (1995). Tuberculosis of the breast masquerading as carcinoma: A study of 100 patients. World J Surg.

[20] Elmrabet F, Ferhati D, Amenssag L, Kharbach A, Chaoui A (2002). Breast tuberculosis. Med Trop (Mars).

[21] Tewari M, Shukla HS (2005). Breast tuberculosis: Diagnosis, clinical features and management. Indian J Med Res.

[22] Shukla US, Kumar S (1989). Benign breast disorders in nonwestern populations: Part U - Benign breast disorders in India. World J Surg.

[23] McKeown KC, Wilkinson KW (1952). Tuberculosis of the breast. Br J Surg.

[24] Dubey MM, Agravval S (1968). Tuberculosis of the breast. J Indian Med Assoc.

[25] Vassilakos P (1973). Tuberculosis of the breast: Cytological findings with fine-needle aspiration, A case clinically and radiologically mimicking carcinoma. Acta Cytol.

[26] Domingo C, Ruiz J, Roig J, Texido A, Aguilar X, Morera J (1990). Tuberculosis of the breast: A rare modern disease. Tubercle.

[27] Kakkar S, Kapila K, Singh MK, Verma K (2000). Tuberculosis of the breast: A cytomorphologic study. Acta Cytol.

[28] Gupta D, Rajwanshi A, Gupta SK, Nijhawan R, Saran RK, Singh R (1999). Fine needle aspiration cytology in the diagnosis of tuberculous mastitis. Acta Cytol.

[29] Martinez-Parra D, Nevado-Santos M, Melendez-Guerrero B, García-Solano J, Hierro-Guilmain CC, Pérez-Guillermo M (1997). Utility of fine needle aspiration in the diagnosis of granulomatous lesions of the breast. Diagn Cytopathol.

[30] Mehrotra R (2004). Fine needle aspiration diagnosis of tuberculous mastitis. Indian J Pathol Microbiol.

[31] Gupta R, Gupta AS (1982). Tubercular mastitis. Int Surg.

[32] Lilleng R, Paksay N, Vural C, Langmark F, Hagmar B (1995). Assessment of fine needle aspiration cytology and histo-pathology for diagnosing male breast masses. Acta Cytol.

[33] Woods GL (1999). Molecular methods in the detection and identification of mycobacterial infections. Arch Pathol Lab Med.

[34] Catanzaro A, Perry S, Clarridge JE, Dunbar S, Goodnight-White S, LoBue PA (2000). The role of clinical suspicion in evaluating a new diagnostic test for active tuberculosis: Results of a multicenter prospective trial. JAMA.

[35] (1997). Rapid diagnostic tests for tuberculosis: What is the appropriate use? American Thoracic Society Workshop. Am J Respir Crit Care Med.

[36] Carpentier E, Drouillard B, Dailloux M, Moinard D, Vallee E, Dutilh B (1995). Diagnosis of tuberculosis by Amplicor Mycobacterium tuberculosis test: A multicenter study. J Clin Microbiol.

[37] (1997). Case definitions for infectious conditions under public health surveillance. MMWR Recomm Rep.

[38] Wobeser WL, Krajden M, Conly J, Simpson H, Yim B, D'costa M (1996). Evaluation of roche amplicor PCR assay for mycobacterium tuberculosis. J Clin Microbiol.

[39] Cohen RA, Muzaffar S, Schwartz D, Bashir S, Luke S, McGartland LP (1998). Diagnosis of pulmonary tuberculosis using PCR assays on sputum collected within 24 hours of hospital admission. Am J Respir Crit Care Med.

[40] Lima DM, Colares Jk, da Fonseca BA (2003). Combined use of the polymerase chain reaction of adenosine activity on pleural fluid improves the rate of diagnosis of plural tuberculosis. Chest.

[41] Villegas MV, Labrada LA, Saravia NG (2000). Evaluation of polymerase chain reaction, adenosine deaminase and interferon-gamma in pleural fluid for the differential diagnosis of pleural tuberculosis. Chest.

[42] Kibiki GS, Mulder B, van der Ven AJ, Sam N, Boeree MJ, van der Zanden A (2007). Laboratory diagnosis of pulmonary tuberculosis in TB and HIV endemic settings and the contribution of real time PCR for M tuberculosis in bronchoalveolar lavage fluid. Trop Med Int Health.

[43] Wiener RS, Della-Latta P, Schluger NW (2005). Effect of nucleic acid amplification for mycobacterium tuberculosis on clinical decision making in suspected extrapulmonary tuberculosis. Chest.

[44] Hasaneen NA, Zaki ME, Shalaby HM, El-Morsi AS (2003). Polymerase chain reaction of pleural biopsy is a rapid and sensitive method for the diagnosis of tuberculous pleural effusion. Chest.

[45] Shah S, Miller A, Mastellone A, Kim K, Colaninno P, Hochstein L (1998). Rapid diagnosis of tuberculosis in various biopsy and body fluid specimens by the AMPLICOR Mycobacterium tuberculosis polymerase chain reaction test. Chest.

[46] Ruiz-Manzano J, Manterola JM, Gamboa F, Calatrava A, Monsó E, Martínez C (2000). Detection of mycobacterium tuberculosis in paraffin-embedded pleural biopsy specimens by commercial ribosomal RNA and DNA amplification kits. Chest.

[47] Vlaspolder F, Singer P, Roggeveen C (1995). Diagnostic value of an amplification method (Gen-Probe) compared with that of culture for the diagnosis of tuberculosis. J Clin Microbiol.

[48] Jawahar MS (2004). Current trends in chemotherapy of tuberculosis. Indian J Med Res.

[49] Khurram M, Tariq M, Shahid P (2007). Breast cancer with associated granulomatous axillary lymphadenitis: A diagnostic and clinical dilemma in regions with high prevalence of tuberculosis. Pathol Res Pract.

[50] Akçay MN, Sağlam L, Polat P, Erdoğan F, Albayrak Y, Povoski SP (2007). Mammary tuberculosis-importance of recognition and differentiation from that of a breast malignancy: Report of three cases and review of the literature. World J Surg Oncol.

[51] Makanjuola D, Murshid K, Al Sulaimani S, Al Saleh M (1996). Mammographic features of breast tuberculosis: The skin bulge and sinus tract sign. Clin Radiol.

[52] Khanna R, Prasanna GV, Gupta P, Kumar M, Khanna S, Khanna AK (2002). Mammary tuberculosis: Report on 52 cases. Postgrad Med J.

[53] Al-Marri MR, Aref E, Omar AJ (2005). Mammographic features of isolated tuberculous mastitis. Saudi Med J.

[54] Kumar P, Sharma N (2003). Primary MDR-TB of the breast. Indian J Chest Dis Allied Sci.

[55] Elsiddig KE, Khalil EA, Elhag IA, Elsafi ME, Suleiman GM, Elkhidir IM (2003). Granulomatous mammary disease: Ten years experience with fine needle aspiration cytology. Int J Tuberc Lung Dis.

